# Supplementary material for: The Stage-Specific Plasticity of Descending Modulatory Controls in a Rodent Model of Cancer-Induced Bone Pain
Source: Cancers (Basel). 2020 Nov 6;12(11):3286. doi: 10.3390/cancers12113286 (PMC7716240; doi:10.3390/cancers12113286)
Supplement: Supplementary file 1 [file cancers-12-03286-s001.zip › supplementary/supplementary.pdf]

cancers

MDPI

Supplementary

The stage specific plasticity of descending modulatory controls in a rodent model of cancer induced bone pain

Mateusz Wojciech Kucharczyk [1,2,*], Diane Derrien [2], Anthony Henry Dickenson[2] and Kirsty Bannister [1]

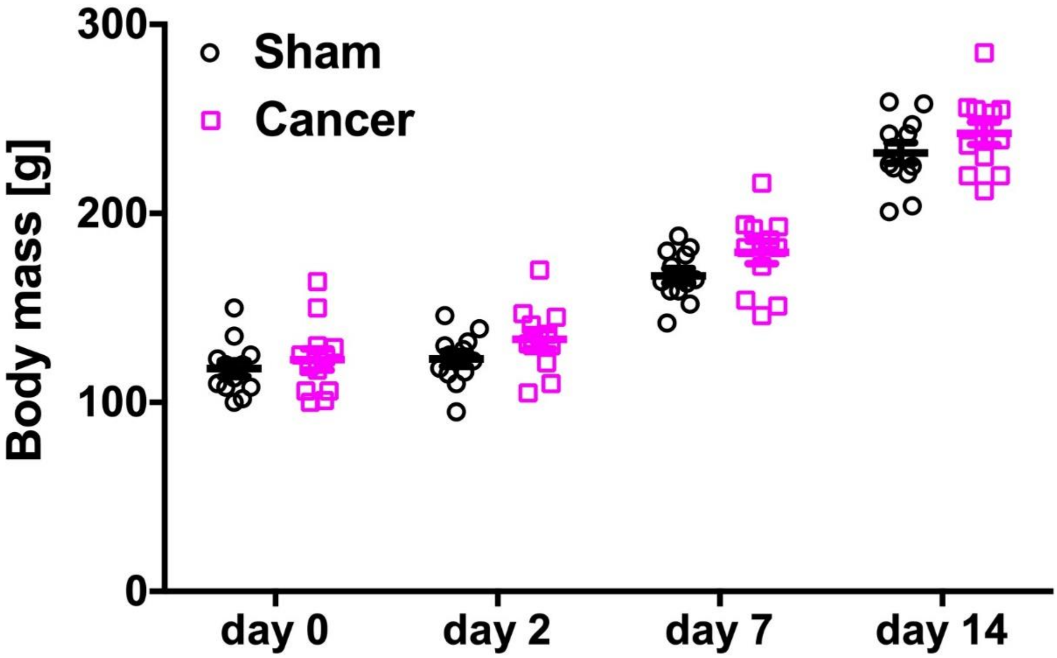

Figure S1. Body mass gain in rats after cancer implantation to tibia is shown. Within a timepoint, each dot represents a single animal (n = 12 per group). Data represent the mean ± SEM. Kruskal-Wallis H for independent samples: all non-significant.

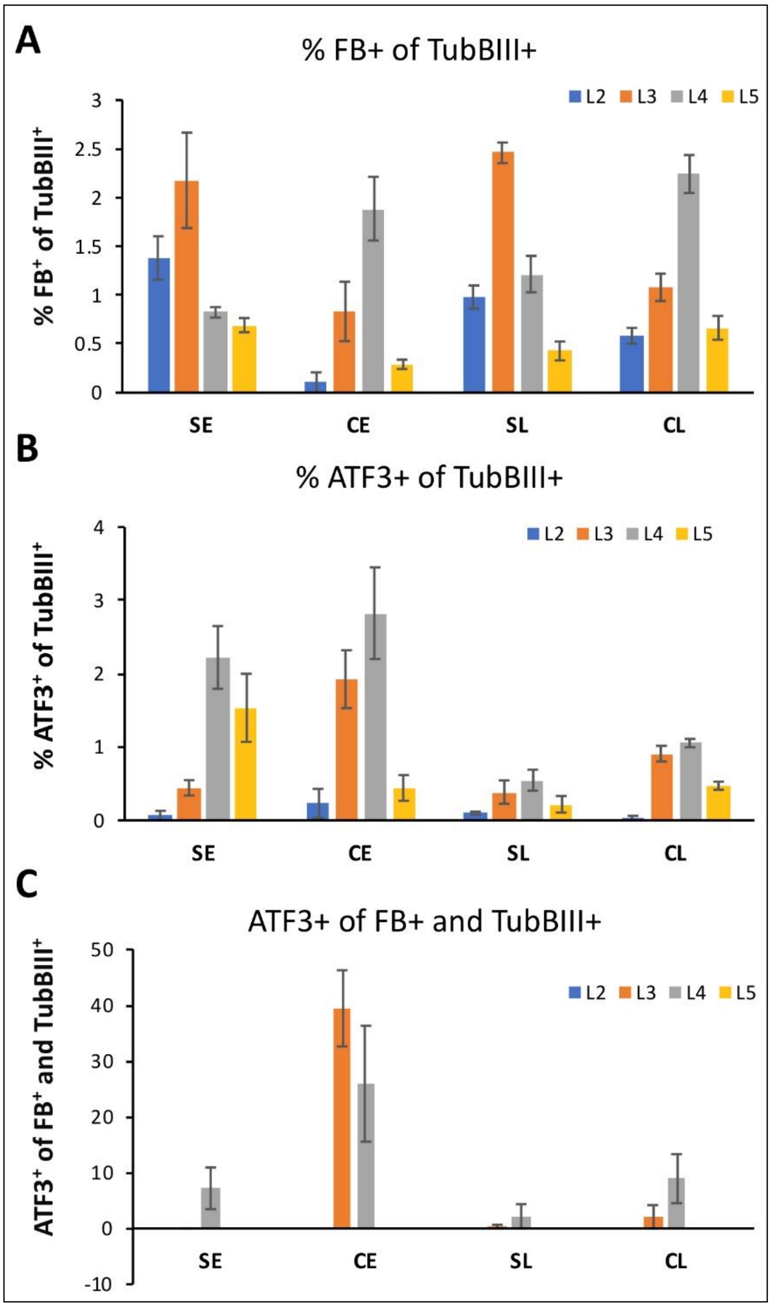

Figure S2. Detailed Atf3 quantification in separate dorsal root ganglia. (A) Total number of Fast Blue (FB) traced tibial afferents within ipsilateral L2-5 DRG analysed as a percentage of all neurons (Tubullin-βIII) therein. On average 100.6 15.7 L2-5 DRG neurons innervate tibia. No FB positivity was noticed in the contralateral lumbar DRG (not shown). **(B)** Quantification of all Atf3[+] afferents within ipsilateral L2-5 DRG, analysed as a percentage of all neurons (Tubullin-βIII) therein in cancer early (CE, day 7/8) and cancer late (CL, day 14/15) stage groups with corresponding sham groups (early – SE and late – SL). **(C)** Quantification of Atf3+ afferents within ipsilateral L2-5 DRG analysed as a percentage of all FB traced neurons. On average 4-20 10 µm sections were counted per DRG. Data represent the mean ± SEM (n = 3, 'n' denotes a separate animal).

© 2020 by the authors. Submitted for possible open access publication under the terms and conditions of the Creative Commons Attribution (CC BY) license (http://creativecommons.org/licenses/by/4.0/).
